# The current state of knowledge on imaging informatics: a survey among Spanish radiologists

**DOI:** 10.1186/s13244-022-01164-0

**Published:** 2022-03-02

**Authors:** Daniel Eiroa, Andreu Antolín, Mónica Fernández del Castillo Ascanio, Violeta Pantoja Ortiz, Manuel Escobar, Nuria Roson

**Affiliations:** 1 Department of Radiology, Institut de Diagnòstic per la Imatge (IDI), Hospital Universitari Vall d’Hebron, Passeig de la Vall d’Hebron, 119-129, 08035 Barcelona, Spain; 2 Department of Radiology, Hospital Universitario Nuestra Señora de Candelaria, Ctra. Gral. del Rosario, 145, 38010 Tenerife, Spain

**Keywords:** Artificial intelligence, Medical informatics, Medical education, Surveys and questionnaires, Radiology

## Abstract

**Background:**

There is growing concern about the impact of artificial intelligence (AI) on radiology and the future of the profession. The aim of this study is to evaluate general knowledge and concerns about trends on imaging informatics among radiologists working in Spain (residents and attending physicians). For this purpose, an online survey among radiologists working in Spain was conducted with questions related to: knowledge about terminology and technologies, need for a regulated academic training on AI and concerns about the implications of the use of these technologies.

**Results:**

A total of 223 radiologists answered the survey, of whom 76.7% were attending physicians and 23.3% residents. General terms such as AI and algorithm had been heard of or read in at least 75.8% and 57.4% of the cases, respectively, while more specific terms were scarcely known. All the respondents consider that they should pursue academic training in medical informatics and new technologies, and 92.9% of them reckon this preparation should be incorporated in the training program of the specialty. Patient safety was found to be the main concern for 54.2% of the respondents. Job loss was not seen as a peril by 45.7% of the participants.

**Conclusions:**

Although there is a lack of knowledge about AI among Spanish radiologists, there is a will to explore such topics and a general belief that radiologists should be trained in these matters. Based on the results, a consensus is needed to change the current training curriculum to better prepare future radiologists.

## Key points

- Spanish radiologists desire to delve deeper into imaging informatics.
- Patient safety and adaptation to new technologies are the main concerns.
- A change on radiology education is needed to include artificial intelligence.

## Introduction

There is no doubt that the upsurge of machine learning (ML) and deep learning (DL) algorithms paired with the high amount of digital data generated in radiology is changing this medical specialty. ML is already used in different imaging modalities such as CAD (computer-aided design) systems for breast cancer screening on mammography [1] or nodule detection on thoracic CT or radiography [2]. DL algorithms, in particular convolutional networks, are a promising technique for processing medical imaging data not only in tasks like image classification, object detection, segmentation or registration [3], but also on dose optimization, creation and maintenance of biobanks and structured reporting among others [4].

More than 50,000 articles are returned when the search “Radiology” AND “Artificial Intelligence” OR “Deep Learning” OR “Machine Learning” is done in Pubmed medical research engine, with a “quasi-exponential” slope for the last 10 years. Such is the concern that both the European Society of Radiology (ESR) and the Radiological Society of North America (RSNA) have their own specialized Internet portals dedicated to artificial intelligence (AI) [5, 6], and the latter has even published a peer-reviewed journal fully dedicated to it [7].

For this reason, there is growing concern among radiologists about the future of the profession. Some believe that radiologists will become obsolete in a few years and others, such as the aforementioned societies [4, 8], have a more conservative stance in which AI will enhance the role of the radiologist and turn the job from volume-based to a value-based [9]. Regardless of particular opinions, the irruption of AI in the radiological field, as well as its progressive integration into clinical practice, will bring a radical change in radiology as we currently know it.

The aim of this study is to evaluate general knowledge and concerns about trends on imaging informatics among radiologists currently working in Spain (both residents and attending physicians). All those respondents who had completed residency at the time of the survey are referred to as attending physicians throughout the text.

## Methods

An online survey (Google Forms©, https://forms.gle/Ha8Rbm9yEG2ZCmq29) was designed by the authors formulating 20 questions related to the level of knowledge about trending terminology and technologies according to the most recent and relevant literature [4, 8, 10], the need for a regulated academic training, as well as concerns about the implications of the widespread use of these technologies in the clinical setting, both ethics- and workforce-related. A summary of the survey is displayed in Table 1.

**Table 1:** Questions about the state of knowledge on imaging informatics and concerns, translated to English

**Demographic information**			
Region of the country		17 options	
Gender		Male	
		Female	
		I rather not say	
Are you a resident or attending physician (AP)?		Resident physician	
		Attending physician	
*Only for residents*		*Only for AP*	
Which year of your residency are you currently in?	R1	What is your work setting?	Only public health care
	R2		Only private health care
	R3		Both, mainly public health care
	R4		Both, mainly private health care
Upon finishing your residency, in which setting do you wish to practice your specialty?	I haven't decided yet		Other (explain)
	Both, mainly in public health care	Without taking your residency into consideration, how many years of professional experience do you currently have?	0–5
	Only public health care		6–10
	Only private health care		11–20
	Both, mainly in private health care		21–30
	Other (explain)		> 30
**Technologies and terminology**			
Choose your level of familiarity with the following terms from the provided options:			
*Term*		*Possible answers*	
Artificial intelligence		I do not know this term I have heard or read about this term I have used it professionally on occasion I usually use it professionally	
Algorithm			
Backpropagation			
Blackbox			
Convolutional neural network			
Machine learning			
Python (programming language)			
R (programming language)			
Pandas (Python library)			
PyTorch			
TensorFlow			
**Academic training**			
Do you consider practicing radiology to be routine work?		Yes	
		No	
		Other (explain)	
Do you consider you should pursue academic training in IT and new technologies (artificial intelligence, machine learning, programming, etc.)?		Yes	
		No	
Do you consider said skills and competencies should be included in the specialty's academic program?		Yes	
		No	
		Other (explain)	
Do you consider there is enough time in four years of academic training to include said skills and competencies?		Yes	
		No	
Who do you consider should cover the economic cost of this academic training?		Yourself	
		The organization which you work for	
		Pharmaceutical companies	
		Professional societies	
		Technological companies	
		Other (explain)	
**In the hypothetical case of massive adoption of AI in the field of radiology, how much do the following worry you?**			
*Questions*		*Possible answers*	
Lack of work		1. Not concerned at all	
Increase in workload		2. Not very concerned	
Patient safety		3. Indifferent	
Reduced remuneration per report		4. Concerned	
Adapting to new technologies		5. Very concerned	
**Journals and Congresses/Meetings**			
How many articles on the matter discussed in this survey have you read in the past year?		None	
		1–3	
		4–6	
		7–10	
		11–15	
		> 15	
How many presentations at Congresses on the matter discussed in this survey have you attended?		None	
		1–3	
		4–6	
		7–10	
		11–15	
		> 15	

*AP* attending physician

A link to the survey was distributed among radiologists working in Spain, who were asked to share and publicize it, as widely as possible, among colleagues throughout the country after requesting their permission. It was also shared by some of the regional subsidiaries of the Sociedad Española de Radiología Médica (SERAM). It remained open for 62 days between 30 July and 30 September 2019. Radiologists not working in Spain were excluded.

Responses were stored in a spreadsheet (Google Forms©) that was later transformed into a comma-separated value file that was loaded into a Jupyter notebook using the Python (v 3.4) pandas (v 1.1.4) library for data exploration and statistical analysis. To facilitate analysis and drawing of conclusions, the answers in the Concerns section were grouped into three categories: not concerned (options 1 and 2), indifferent (option 3) and concerned (options 4 and 5). In the instances where group comparison is made between attending physicians and residents, the Chi-square is used (scikit-learn v 0.24.0). Yates’ correction for continuity was applied where necessary. Statistical significance is accepted at *p* < 0.05. Confidence intervals are not provided since the survey was purely descriptive. The results are expressed in percentages of the total answers throughout the manuscript.

## Results

In the spawn of two months, a total of 223 radiologists answered the survey, of whom 171 (76.7%) were attending physicians and 52 (23.3%) residents. When comparing to the current distribution of members in SERAM (836 residents and 5139 nonresidents) [11], we found that we had a greater proportion of residents than expected (*p* < 0.05).

Regarding attending radiologists, 50.9% worked exclusively in the public setting, while 5.8% worked only in the private sector and 38.6% combines public and private dedication. The same proportion (39.2%) had either fewer than 10 years or more than 20 years of working experience.

As per the residents, 44.2% were in their second year of specialty. Upon finishing, 63.5% desire to work in the public setting, mostly with some private dedication (55.8%). 32.7% had not yet decided on their preferred work setting. A summary of the results is shown in Table 2.

**Table 2:** Summary of results

**Demographic information**						
*Gender*						
Female			118/223 (52.9%)			
Male			103/223 (46.2%)			
Rather not say			2/223 (0.9%)			
*Professional level*						
Attending physician			171/223 (76.7%)			
Resident			52/223 (23.3%)			
**Resident section—52 respondents**			**AP section—171 respondents**			
*Year of residency*			*Work setting*			
R1		9/52 (17.3%)	Both, mainly public		66/171 (38.6%)	
R2		23/52 (44.2%)	Both, mainly private		8/171 (4.7%)	
R3		11/52 (21.2%)	Only public		87/171 (50.9%)	
R4		9/52 (17.3%)	Only private		10/171 (5.8%)	
*Desired work setting upon residency completion*			*Experience as AP (in years)*			
Not decided		17/52 (32.7%)	0–5		32/171 (18.7%)	
Both, mainly public		29/52 (55.8%)	6–10		35/171 (20.5%)	
Both, mainly private		2/52 (3.8%)	11–20		37/171 (21.6%)	
Only public		4/52 (7.7%)	21–30		49/171 (28.7%)	
Only private		0/52 (0%)	> 30		18/171 (10.5%)	
**Technologies and terminology**						
	*I do not know this term*	*I have heard or read about this term*	*I have used it professionally on occasion*		*I usually use it professionally*	
Artificial intelligence	2/223 (0.9%)	169/223 (75.8%)	41/223 (18.4%)		11/223 (4.9%)	
Algorithm	17/223 (7.6%)	128/223 (57.4%)	46/223 (20.6%)		32/223 (14.3%)	
Backpropagation	171/223 (76.7%)	45/223 (20.2%)	6/223 (2.7%)		1/223 (0.4%)	
Blackbox	145/223 (65.0%)	73/223 (32.7%)	1/223 (0.4%)		4/223 (1.8%)	
Convolutional neural network	80/223 (35.9%)	131/223 (58.7%)	10/223 (4.5%)		2/223 (0.9%)	
Machine learning	26/223 (11.7%)	167/223 (74.9%)	23/223 (10.3%)		7/223 (3.1%)	
Python	160/223 (71.7%)	56/223 (25.1%)	5/223 (2.2%)		2/223 (0.9%)	
R	181/223 (81.2%)	34/223 (15.2%)	6/223 (2.7%)		2/223 (0.9%)	
Pandas	200/223 (89.7%)	18/223 (8.1%)	3/223 (1.3%)		2/223 (0.9%)	
PyTorch	215/223 (96.4%)	6/223 (2.7%)	1/223 (0.4%)		1/223 (0.4%)	
TensorFlow	181/223 (81.2%)	37/223 (16.6%)	2/223 (0.9%)		3/223 (1.3%)	
**Academic training**						
*Do you consider practicing radiology to be routine work?*						
Yes			82/223 (36.8%)			
No			123/223 (55.2%)			
Other			18/223 (8%)			
*Do you consider you should pursue academic training in IT and new technologies?*						
Yes			223/223 (100%)			
No			0/223 (0%)			
*Do you consider said skills and competencies should be included in the specialty’s academic program?*						
Yes			207/223 (92.9%)			
No			2/223 (2.2%)			
Other			11/223 (4.9%)			
*Do you consider there is enough time in 4 years of academic training to include said skills and competencies?*						
Yes			53/223 (23.8%)			
No			170/223 (76.2%)			
*Who do you consider should cover the economic cost of this academic training? (multiple answer)*						
Yourself			23/223 (10.3%)			
The organization which you work for			188/223 (84.3%)			
Pharmaceutical companies			21/223 (9.4%)			
Professional societies			95/223 (42.6%)			
Technological companies			74/223 (33.2%)			
Other			23/223 (10.3%)			
**Self-education**						
	*None*	*1*–*3*	*4*–*6*	*7*–*10*	*11*–*15*	> *15*
Lectures attended last year	91/223 (40.8%)	93/223 (41.7%)	29/223 (13.0%)	4/223 (1.8%)	2/223 (0.9%)	4/223 (1.8%)
Articles read last year	58/223 (26.0%)	111/223 (49.8%)	30/223 (13.5%)	15/223 (6.7%)	2/223 (0.9%)	7/223 (3.1%)
**Concerns**						
	*Not concerned*		*Indifferent*		*Concerned*	
Lack of jobs	102/223 (45.7%)		65/223 (29.2%)		56/223 (25.1%)	
Workload increase	90/223 (40.4%)		65/223 (29.1%)		68/223 (30.5%)	
Reduced per-report remuneration	47/223 (21.1%)		55/223 (24.7%)		121/223 (54.2%)	
Patient safety	62/223 (27.8%)		49/223 (22.0%)		112/223 (50.2%)	
Adapting to new technologies	71/223 (32.0%)		53/223 (23.8%)		98/223 (44.2%)	

*AP* attending physician

**Fig. 1:**
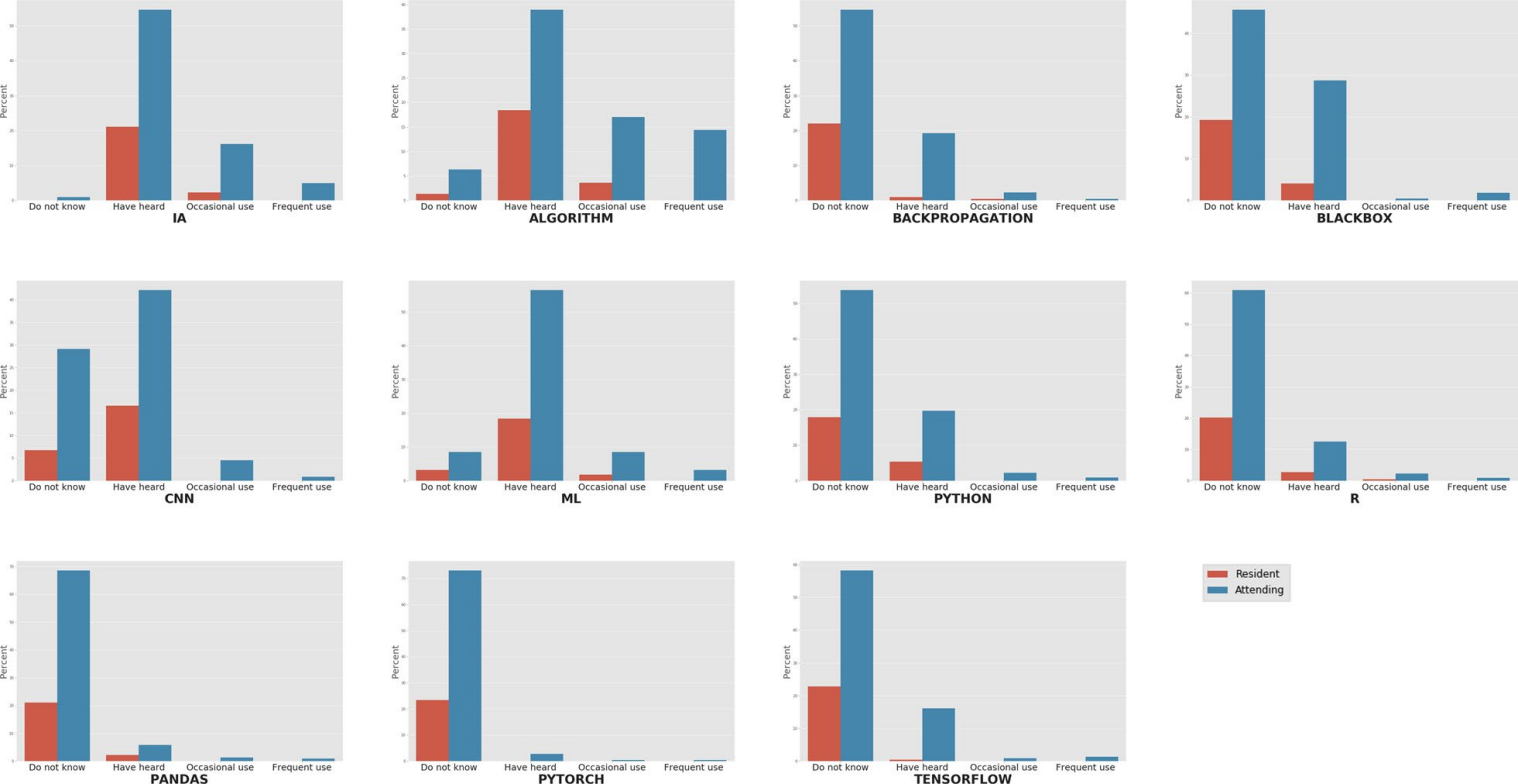
Level of knowledge or usage of the different terms and technologies. Responses are divided into residents (red) and attending physicians (blue). The *y*-axis shows the percentage of answers for each group

With respect to the terminology and technologies, most of the underlying technologies used in deep learning remain unknown to the survey participants, such as Python (71.7%), R (81.2%), PyTorch (96.4%) and TensorFlow (81.2%). Conversely, general terms like artificial intelligence and algorithm had been heard of or read in at least 75.8% and 57.4% of the cases, respectively (Fig. 1). Statistical significance was found for AI, algorithm, backpropagation, blackbox and TensorFlow (Table 3). According to Pearson's residuals, this significance is mainly due to a higher proportion of residents showing occasional use or knowledge only.

**Table 3:** Chi-square analysis of terminology and technologies section

Term	Residents. *n* = 52			Attending physicians. *n* = 171				*p* value
	DK (%)	HH (%)	OU (%)	DK (%)	HH (%)	OU (%)	FU (%)	
Artificial intelligence	0.0	90.4	9.6	1.2	71.3	21.1	6.4	0.03*
Algorithm	5.8	78.8	15.4	8.2	50.9	22.2	18.7	< 0.001*
Backpropagation	94.2	3.8	1.9	71.3	25.1	2.9	0.6	0.01*
Blackbox	82.7	17.3	0.0	59.6	37.4	0.6	2.3	0.02*
Convolutional neural network	28.8	71.2	0.0	38.0	55.0	5.8	1.2	0.10
Machine learning	13.5	78.8	7.7	11.1	73.7	11.1	4.1	0.41
Python	76.9	23.1	0	70.2	25.7	2.9	1.2	0.48
R	86.5	11.5	1.9	79.5	16.4	2.9	1.2	0.66
Pandas	90.4	9.6	0.0	89.5	7.6	1.8	1.2	0.63
PyTorch	100.0	0.0	0.0	95.3	3.5	0.6	0.6	0.47
TensorFlow	98.1	1.9	0.0	76.0	21.1	1.2	1.8	0.01*

*DK* do not know, *HH* have heard, *OU* occasional use, *FU* frequent use. Statistically significant results are marked (*). *Note* no “frequent use” responses for the resident group

**Fig. 2:**
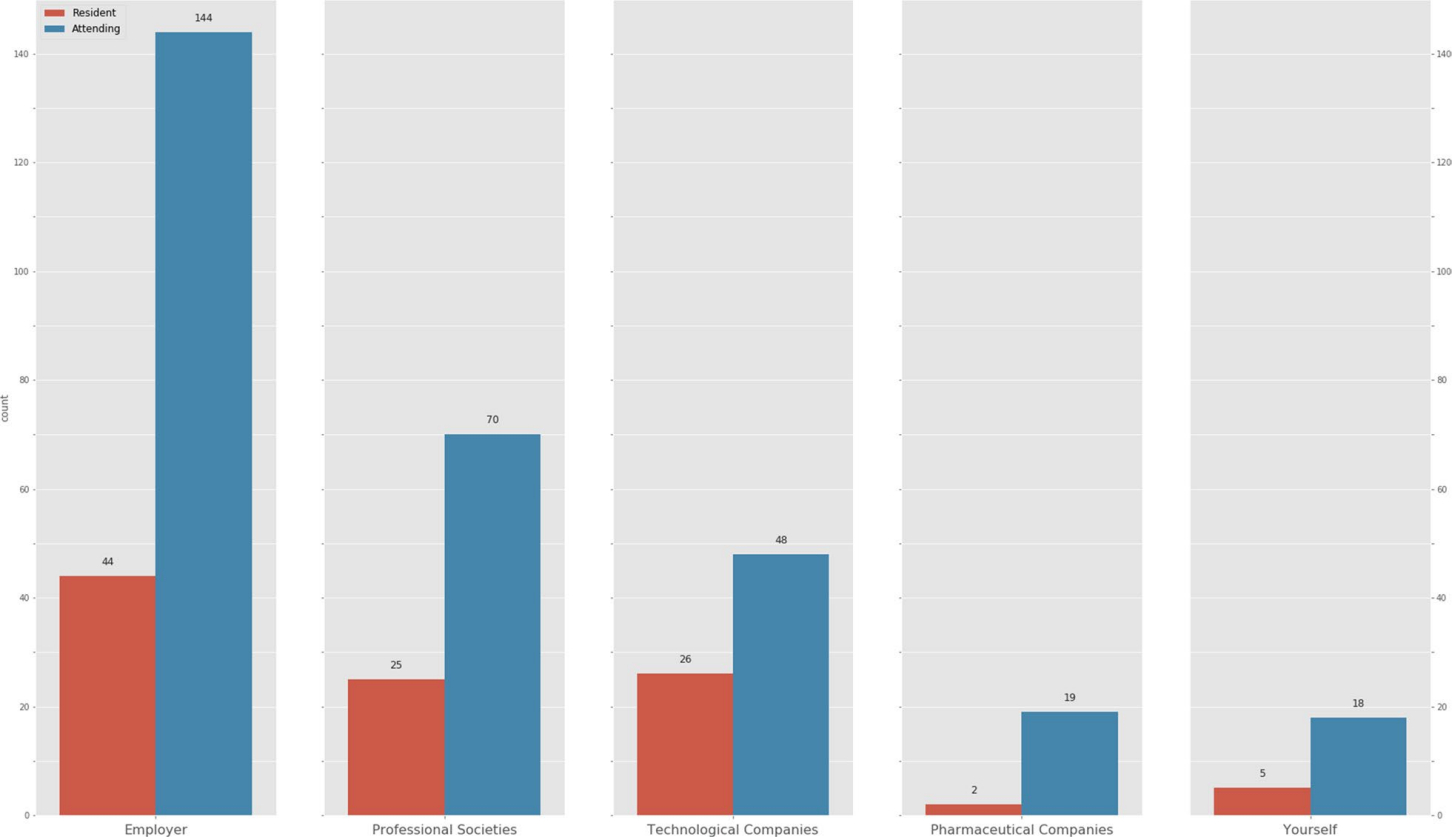
Distribution of responses about who should financially cover the costs of AI training. Responses are divided into residents (red) and attending physicians (blue). The *y*-axis shows the number of answers for each group

All the respondents (100%) recognize that they should pursue academic training in medical informatics and new technologies. These skills and competencies should be incorporated in the training program of the specialty according to 92.9%, although 76.8% reckon that there is no time during the 4 years Spanish residency period to include them. Most of the respondents (84.3%) consider that this training should be financially covered by the employing organization (Fig. 2). No significant differences were observed between groups, except for technological companies, chosen by a bigger proportion of residents (50% vs 28.1%, *p* = 0.006) (Table 4).

**Table 4:** Chi-square analysis of cost of training section

Who should pay?	Residents *n* = 52 (%)	AP *n* = 171 (%)	*p* value
Technological companies	50	28.1	0.006*
Yourself	9.6	10.5	0.85
The organization which you work for	84.6	84.2	0.94
Pharmaceutical companies	3.8	11.1	0.19
Professional societies	48.1	40.9	0.45

*AP* attending physician. Statistically significant results are marked (*)

**Fig. 3:**
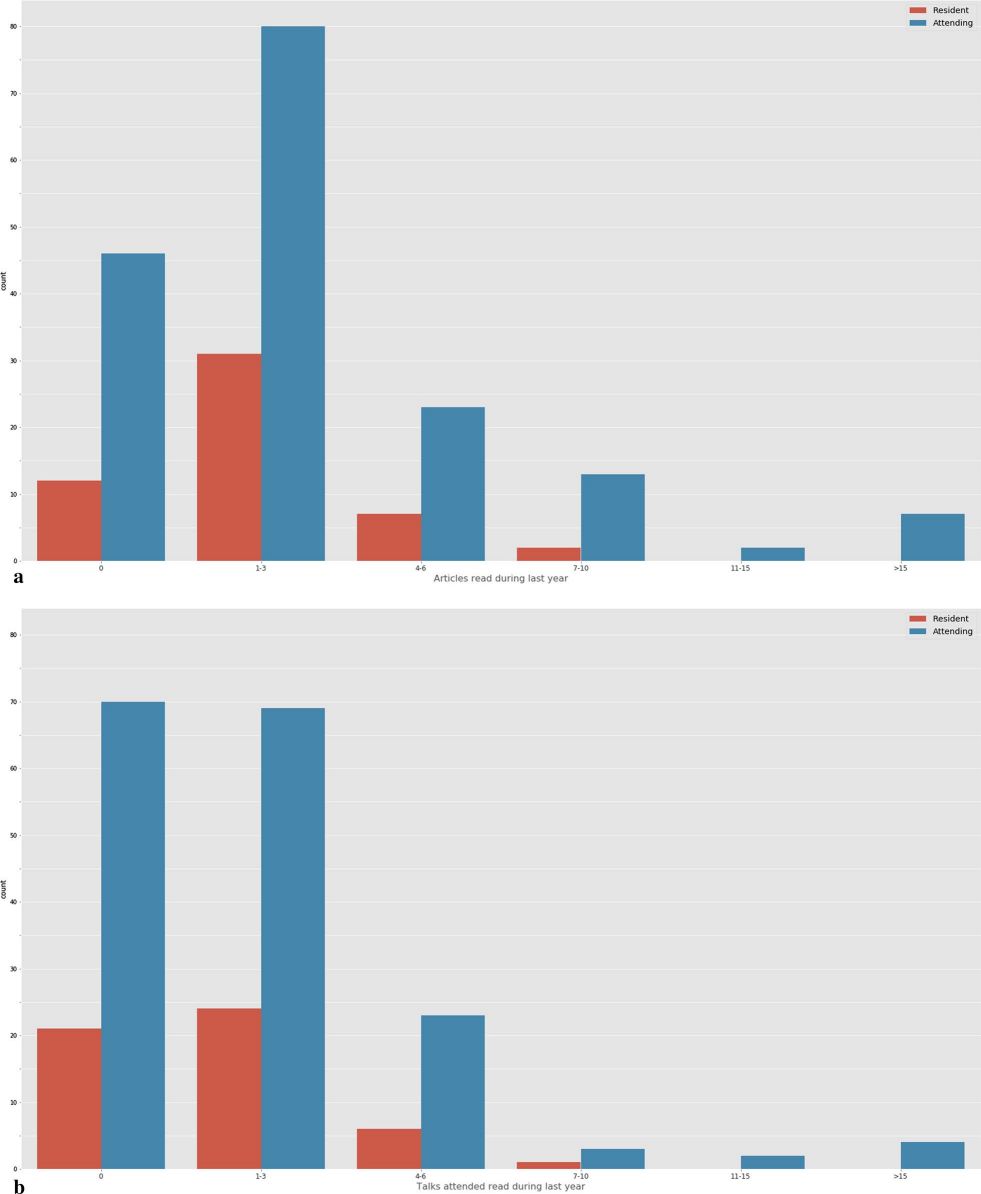
Articles read (**a**) and lectures attended (**b**) during the year prior to the survey. Responses are divided into residents (red) and attending physicians (blue)

40.8% of the respondents had not attended to any communications or lectures on the topic during the previous year, while 41.7% had attended to less than three. Similarly, 49.8% of the radiologists had read between one and three and 26% had not read any scientific articles on the subject (Fig. 3).

**Fig. 4:**
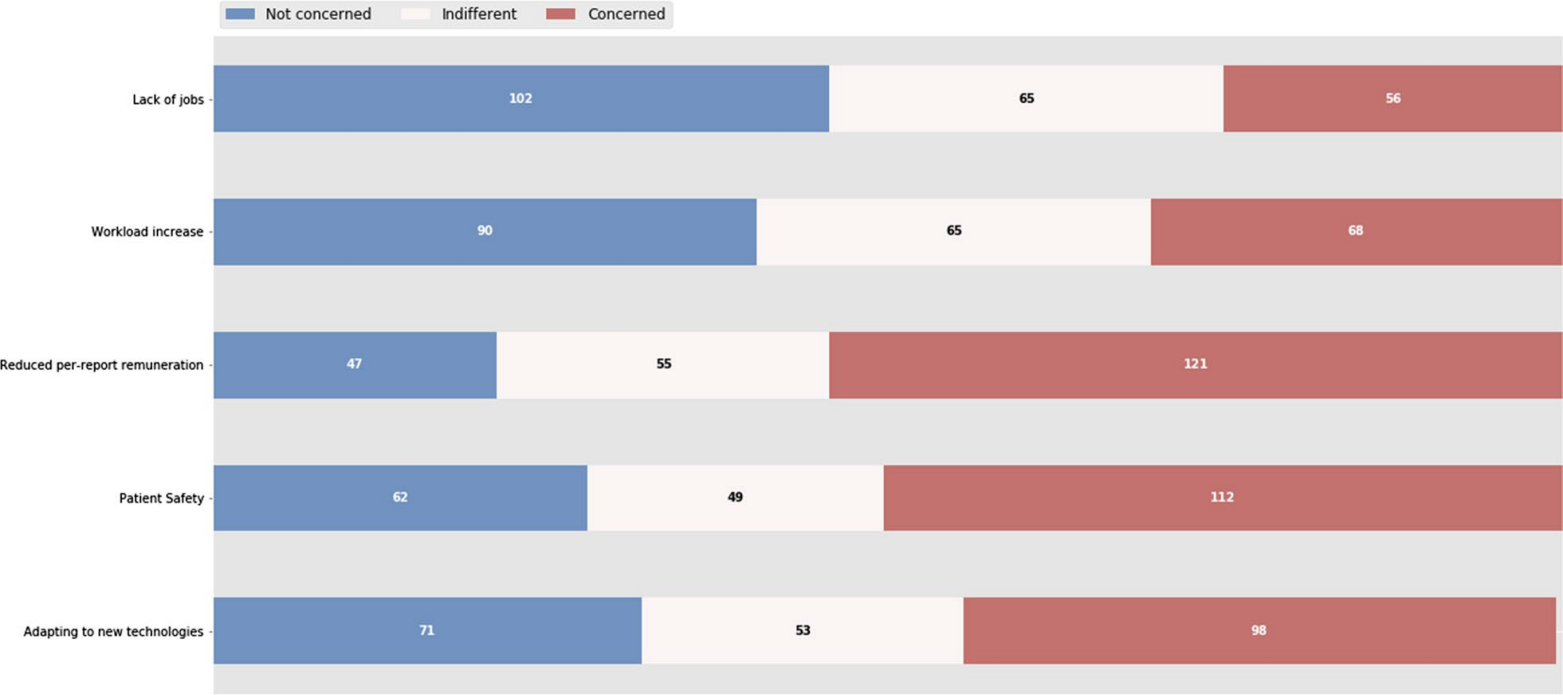
Concerns about the adoption of AI in radiology, as perceived by the respondents. The *x*-axis shows the number of answers

Patient safety (54.2%), adaptation to new technologies (50.2%) and a reduced per-report retribution (44.2%) were the main concerns. On the other hand, job loss was not seen as a peril by 45.7% of the participant. Regarding workload increase, 40.4% were not worried while 30.5% manifested some concern (Fig. 4).

## Discussion

We have assessed the general understanding and concerns on AI-related topics among radiologists working in Spain. Similar studies have been carried out in the past years in other European countries such as Italy [12], France [13] or Switzerland [14], and even among ESR members [15]. There have also been surveys in the USA [16], Singapore [17] and Saudi Arabia [18]. Perhaps one of the most comprehensive studies in this regard so far is the two-part international survey conducted by Huisman et al. [19, 20] in 2019.

Our distribution of respondents showed a greater proportion of residents than expected based on the SERAM members distribution. This trend was also seen in a nationwide online Italian survey responded by 1032 *Società Italiana di Radiologia Medica e Interventistica* members, in which the age distribution of responders was younger than expected [12]. There could be a great variety of factors to explain our results such as a greater interest in AI among younger radiologists since it might have a greater impact in their future career, a belief also shared by the authors of the Italian survey [12]. Nevertheless, sample size or accessibility to the survey could have also played a part. For instance, the circulation of our survey was by convenience, generating an inherent bias based on whoever received the survey and their contacts.

When asked about AI and specific terms the vast majority have either heard about AI or used it in daily work while only 2 of the 223 respondents have not heard about AI. This result is consistent with other similar surveys [16–20]. Other generic terms such as algorithm, ML or convolutional neural network were also familiar to the 50–75% of participants. This is to be expected as in the recent years there has been an outburst of lectures and articles about AI, some radiological societies such as ESR and Canadian Association of Radiology have redacted white papers on this topic [4, 21], and others, such as EuSoMII [22], an institutional member society of the ESR, conduct training activities on AI.

However, when asked about more specific terms such as backpropagation or blackbox or programming languages like Python or R, the results were reversed and most of the participants had little clue about them. The significant differences found among groups for terms such as AI, algorithm, backpropagation and blackbox could be explained by a higher exposure or use of commercial products by the attending physicians or by their involvement in research projects. Collado-Mesa et al. [16] found that only 33% of the respondents from a survey in a single center in the USA recognized speech recognition as an AI tool even though all of the participants used it on a daily basis. Our results are also in line with the work of Huisman et al., in which only a minority of respondents (16%) have advanced AI skills [19]. This manifests that although radiologists are aware of AI, most of them only scratch the surface or are unaware that AI/ML is already implemented. If that alone is enough to survive in a future in which AI is fully operational in the daily workflow is yet to be seen. In fact, in our survey 44.2% of the participants were concerned about adapting to the new technologies as opposed to the 32% that thought otherwise.

The aforementioned could be associated with the number of AI/ML-based lectures or papers read in the past year. Overall, 82.5% attended to three or less lectures (40.8% none) and 75.8% read three or less papers (26% none). A multicenter survey conducted in Singapore [17] showed similar results in which 29.6% of participants had not read a single paper in the previous six months and 56% between one and five. Similarly, 80.8% did not attend any data science course in the previous 5 years [17]. Collado-Mesa et al. [16] argued in 2017 that a possible explanation for the low exposure to AI/ML scientific literature could be the relatively few AI-based articles in main radiology journals, although we believe this is drastically changing as the number of articles is growing exponentially. Another reason for this low exposure might be the lack of implementation of AI/ML programs in residency. For instance, there is no mention of image computing in the Spanish official radiology residency curriculum other than a reference to office software and teleradiology tools as well as the use of the Internet as an information source [23]. As for the European training curriculum, the Level I and II document (March 2020) cites “to understand functioning and application of AI tools, knowledge of ethics of AI and performance assessment and critical appraisal” [24].

Every participant manifested the need to pursue academic training in new technologies and 92.9% think this should be implemented in the specialty’s academic program. This is also stated in other similar surveys [15–17, 20].

In this regard, Lindqwister et al. [25] conducted a recent pilot model for an integrated AI curriculum (AI-RADS) in radiology in Dartmouth, USA. The course was based on a sequence of foundational algorithms in AI presented as logical extensions of each other in lessons of one hour (once per month for a total of seven months) and reinforced with a concurrent journal club highlighting the algorithm discussed in the previous lecture. The course was also paired with secondary lessons in key topics such as pixel mathematics since most of the participants did not have a computational background. The course received a 9.8/10 satisfaction and residents perceived better understanding of basic concepts in AI [25].

Wiggings et al. [26] designed and implemented a focused data science pathway for senior radiology residents. In this model, three fourth-year residents with varying technical background were involved in a data science pathway aimed to address all stages of clinical ML model development (fundamentals, data curation, model development and clinical integration) with proper mentorship. The authors noted that fundamentals in mathematics, basic coding, ML theory, data curation and model development as well as clinical integration are key factors for a successful engagement in clinical data science. The experience gained in this pilot project would help improve the training experience in consecutive years for a long-term success of this curriculum. At the end of the training, participants showed a desire for a more formal didactic curriculum [26].

A pilot project for residents including skills such as data management, general ML and DL using Python is being conducted at our institution, with emphasis on evaluation and critical assessment. After this pilot experience is completed and evaluated, we plan to implement a formal program for all the second-year (basic knowledge and theoretical approach) and third- and fourth-year residents (hands-on project-based approach), with guidance and mentorship from the first cohort of residents.

There was a consensus among our respondents that adding an AI curriculum into the 4-year residency program might not be enough for a good formation in AI. These results are difficult to interpretate since the required level or knowledge of that training was not included in the questionary, and neither was asked whether participants were aware of the European training curriculum previously mentioned. To the best of our knowledge, there is no consensus in Europe about what this teaching itinerary should be. Regardless, we believe the current model needs to change if we want to produce radiologists with the sufficient AI skills during the radiology residency or beyond that. Trainees might not need to spend that much time mastering pattern recognition and could spend more time learning data science and AI [27].

The general lack of concern about job loss shown in our results is also shared in almost every survey [12, 14, 15, 17–20], in which radiologists are rather keen to incorporate it in daily work. In the Italian survey, about 66% of the respondents defined AI as an aid to daily practice and believed that image interpretation will be handled by AI [12]. There is also a consensus that AI will help reduce errors and optimize radiologists’ work [12, 13] as well as the administrative burden [16]. This is in agreement with the results from the international survey carried on by Huisman et al., in which the responders believe AI will change radiologists tasks rather fully replace them [19]. The most extensive series show a concern for the change in the radiologist's tasks (82%) much more than a total replacement of radiologists (10%) [19]. Recent advances, then, lead us to consider that AI will change radiology as we currently know it. But perhaps the transformation will not be as disruptive as initially imagined, but more organic and intertwined with the daily workflow granting us to achieve a synergy between radiologist and computer, allowing radiologists to spend their time performing value-added functions and increase their professional satisfaction. This view is supported by some of the existing literature, where as many as 77% of respondents believe that workflow optimization will be possible by these tools [20]. In this regard, AI should be viewed as a wingman instead of a direct competitor and we should not be afraid of incorporating AI and view it as an opportunity to be in the vanguard as it might also have an impact in another medical fields. In fact, there is a growing belief that it could empower radiologists creating a new profile focused on data analysis: radiologists as clinical data scientists [28]. There is indeed a need to reshape the current radiologist role to avoid being overtaken by clinicians or by other experts in AI and even lead AI projects involving radiological images. Some of the free answers given by the respondents are in concordance with this statement such as “we need to work with engineers and IT. We are the analysts, and they are the developers.” This goes against some of the most pessimistic opinions, which also do not consider some tasks that cannot be yet performed by AI such as interventionism, both vascular and non-vascular, or participation in multidisciplinary meetings.

Opinions about the workload increase were quite similar, as 30.5% were concerned about a possible increase while 40.4% were not worried at all. In the EuroAIM survey almost 75% of the respondents felt that AI will impact workload, but it was unclear whether it might be an increase or not [15] and in the Italian survey there was a minority who thought that AI would increase workload [12]. Both articles concluded that these differences reflect the doubt on the impact of AI in radiology, a statement we share based on our own results.

The EuroAIM survey showed that 55% of responders believe that patients will not accept a report made by an AI-application alone [15], results that are in line with other publications [3]. Participants in the Swiss survey did not agree on where liability should lie in the event of an AI error [14]. A similar feeling is shared by the most pessimist articles regarding the future of AI, in which the “human barrier” might be an obstacle for the full replacement of radiologists [29]. In our study, 50.2% of the participants were concerned about patient safety presumably due to the ethical–legal implications. Certainly, there is still debate on how errors, discrepancies and malpractice when using these tools will be managed, which may result in reluctancy toward its adoption not only by radiologists but also by patients and other medical fields [29, 30]. Ongena et al. [31] performed a questionnaire to 21 patients in the Netherlands and revealed that they were not optimistic about AI systems taking over diagnostic interpretations performed by radiologists. This highlights the importance of a strict regularization of AI tools in the field of radiology and the pivotal role of the patient in this process.

Finally, the opinion of students is also important as they are the future radiologists. Gong et al. [32] conducted a survey on 322 medical students in Canada revealed that 67.7% of them considered that AI would reduce the demand for radiologists and discouraged them from applying to the radiology specialty. However, prior AI background was associated with a more positive attitude. The Swiss survey also concurred that students might be afraid to specialize in radiology because of the peril of AI being a detrimental factor to their work security [14]. Due to the positive effect of previous AI knowledge in dissipating the fear of AI, we agree with both authors that AI curriculum should be included not only in radiology residency but also during bachelor’s degree in medicine.

We consider the main limitation of our study to be selection bias stemming from both initial survey disclosure and convenience sampling secondary to subsequent sharing by respondents to colleagues and acquaintances. Also, a greater participation would improve the robustness of our results and allow us to draw better conclusions. On retrospective, we found certain methodological flaws on the design of certain questions and options provided, which we consider could be improved in order to better assess the responses. Specifically, the precise reasons about patient safety and ethical–legal implications were not asked, therefore the interpretation of these results may be somewhat ambiguous.

In conclusion, there is a general lack of knowledge about such topics among Spanish radiologists, both members in training and attending physicians. Nevertheless, there is widespread enthusiasm to delve deeper into this matter, as also seen in surveys carried out in other countries. Job loss was not a major concern among our surveyees, against the most ominous voices that predict the extinction of our discipline. While there are a few institutional initiatives, including ours, that aim to train their radiologists in this domain, there is no doubt that a common consensus is needed to change the current training curriculum to prepare new radiologists for a future world in which AI will undoubtedly shape the profession.

## Data Availability

The datasets used and/or analyzed during the current study are available from the corresponding author on reasonable request.

## Abbreviations

AI: Artificial intelligence
AP: Attending physician
CAD: Computer-aided design
DL: Deep learning
ESR: European Society of Radiology
ML: Machine learning
RSNA: Radiological Society of North America
SERAM: Sociedad Española de Radiología Médica
